# Tensductor—Amorphous Alloy Based Magnetoelastic Tensile Force Sensor

**DOI:** 10.3390/s18124420

**Published:** 2018-12-14

**Authors:** Michał Nowicki

**Affiliations:** Institute of Metrology and Biomedical Engineering, Warsaw University of Technology, 02-525 Warsaw, Poland; nowicki@mchtr.pw.edu.pl; Tel.: +48-690-650-386

**Keywords:** force sensor, magnetoelastic sensor, pressductor, amorphous alloy

## Abstract

In this paper new, tensile force sensor is presented, based on Pressductor topology and single layer of ferromagnetic amorphous ribbon. Simplified operating principle of the magnetic core with orthogonal coils is described. Straight and diagonal cut sensors are compared. The load vs. induced voltage characteristics are presented, as well as possibility of higher harmonics utilization. The effect of supply current on signal amplitude and measurement hysteresis is given. The developed ‘Tensductor’ sensor has near-linear characteristics and is relatively easy to manufacture. The measurement range is scalable, the experimental unit had 0–12 N measurement range with 1% accuracy, mostly due to magnetoelastic hysteresis.

## 1. Introduction

**Figure 1:**
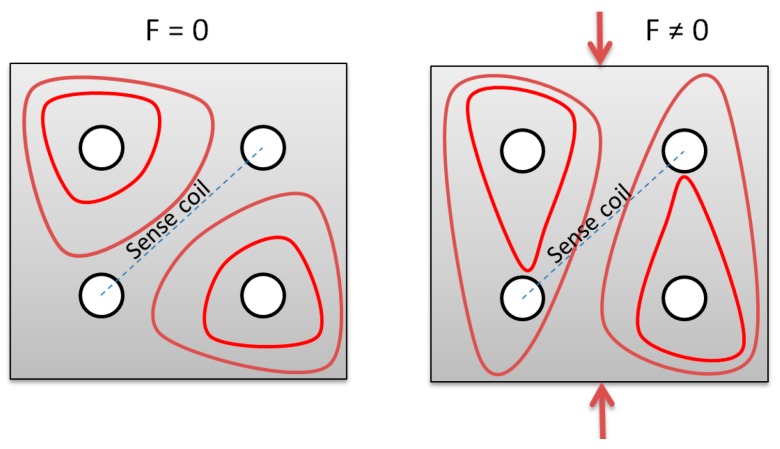
Pressductor operating principle (red lines—magnetic flux lines). Adapted from [1].

There are numerous force sensing techniques [1], among which magnetoelastic effect sensors plays a special role [2]. They are mostly used in harsh industrial conditions, in kN-MN measurement ranges [3]. The most common, industry accepted and successful topology is known as Pressductor, and is in continuous production since 1954 [4]. Schematic diagram of typical Pressductor operating principle is shown in Figure 1.

The Pressductor consists of bulk or laminated steel core, with four holes thru which two perpendicular coils are wound—magnetizing and sensing coil. For ideally isotropic magnetic material, lines of magnetic flux induced by magnetizing coil do not cross the plane of sensing coil, due to the system’s symmetry. Therefore, there is no voltage signal induced in the sensing coil. When the force is applied (Figure 1), stress-induced anisotropy of magnetic permeability [5,6] redirects some flux lines through the sensing coil [7]—the system is no longer symmetrical. Therefore, there is voltage signal induced in the sensing windings, which is a convoluted function of applied stress in the material, sensor geometry and magnetoelastic properties of the given material. Most of the Pressductors work in the compressive stress region, as the name implies.

Typically, Pressductors are made from laminated electrical steel, in a standard transformer fashion. In recent years, there were numerous accounts of magnetoelastic sensors made of materials with better magnetoelastic parameters, such as ferrites [8,9], amorphous [10], and nanocrystalline alloys [11].

Therefore in this paper new sensor is presented, based on successful Pressductor topology and state-of-the-art, commercially available amorphous ribbon material.

## 2. Materials and Methods

### 2.1. New Sensor Construction

**Figure 2:**
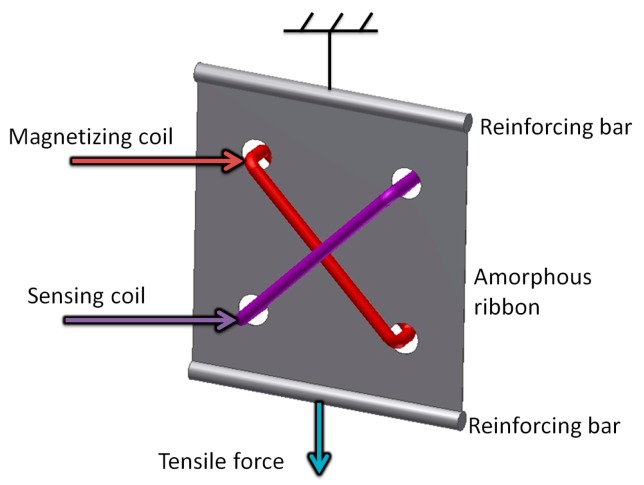
Schematic diagram of tensductor sensor.

The Tensductor schematic, geometry and operating principle follows the original Pressductor. However, due to extremely thin layer of magnetic ribbons available, it is limited to tensile stress sensing. The proposed sensor schematic is presented in Figure 2. For the core, Fe_73.5_Cu_1_Nb_3_Si_15.5_B_7_ amorphous alloy was chosen, in the as-cast state, produced by VACUUMSCHMELZE GmbH & Co. KG (Hanau, Germany). It is a precursor of commercially available nanocrystalline VITROPERM^®^ 800 [12], manufactured using rapid solidification technology. 50 × 50 mm rectangular piece of amorphous ribbon was cut from the alloy tape, and the holes for 10 magnetizing and 10 sensing windings were made with Electrical Discharge Machining. There is also the possibility of photolithographic amorphous or nanocrystalline core preparation [13].

**Figure 3:**
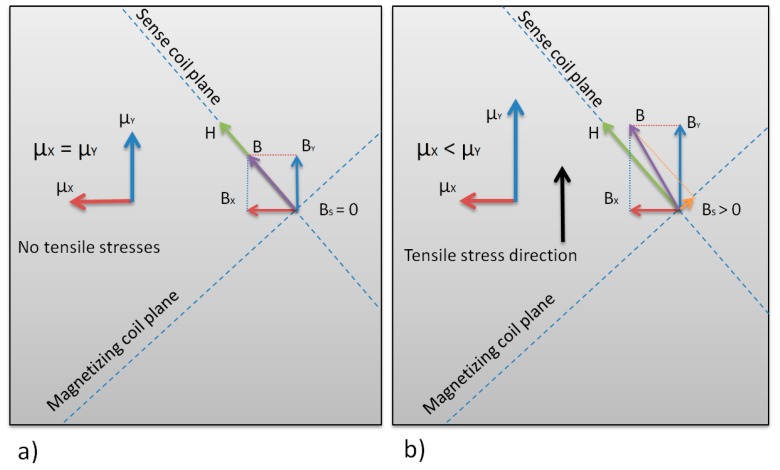
Mechanism of measurement voltage induced in the tensductor sensor. (**a**) No load, and (**b**) loaded.

In the Figure 3 the simplified operating principle of the proposed sensor is presented. For given thin magnetic ribbon, lets analyze the point lying on the intersection of the perpendicular sense coil and magnetizing coil planes, which in turn are rotated 45° in relation to XY coordinates. If there is a magnetizing current in the magnetizing coil, magnetizing field **H** is produced. If we distinguish two perpendicular permeability values in the magnetic material, μ_X_ and μ_Y_, there will also be two magnetic induction vector components, B_X_ and B_Y_, from the simplified equation:

(1) $$B_{i}=\mu_{i}H$$

If the permeabilities μ_X_ = μ_Y_, the resultant vector **B** will be parallel to the sense coil, and hence could not induce any measured voltage (Figure 3a).

If however the permeabilities μ_X_ ≠ μ_Y_, the resultant vector **B** will change direction, and there will be component vector **B**_S_ perpendicular to the plane of the sense coil (Figure 3b).

The **B**_S_ can be calculated from component induction vector values by rotation of the coordinate system axes by 45° [14]:

(2) $$B_{S}=-\frac{\sqrt{2}}{2}B_{X}+\frac{\sqrt{2}}{2}B_{Y}=-\frac{1}{2}\mu_{X}H+\frac{1}{2}\mu_{Y}H$$

as can be seen from (2), if μ_X_ = μ_Y,_ then **B**_S_ = 0. However, the μ_X_ ≠ μ_Y_, and that is the case when there is stress-induced anisotropy of magnetic permeability, the **B**_S_ will induce measurement voltage ε in the sensing winding of area S and n turns according to induction law:

(3) $$\varepsilon =-n\frac{{d\Phi }_{S}}{\mathrm{dt}}=-n\frac{{\mathrm{dB}}_{S}}{\mathrm{dt}}S$$

The measured signal will be therefore higher for higher number of sensing turns, bigger core cross-section, higher magnetizing frequency, and higher difference between μ_X_ and μ_Y_.

**Figure 4:**
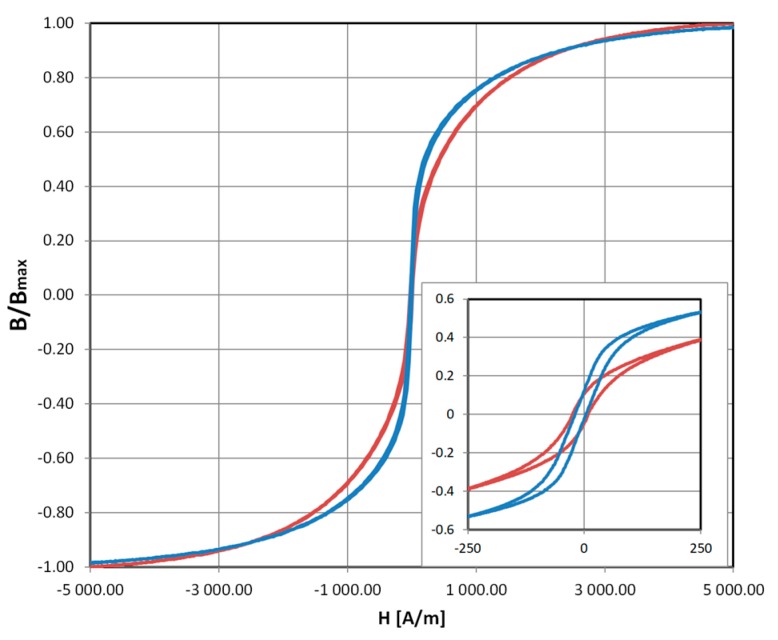
Magnetic B(H) hysteresis loops for the sensor material in as-cast state. Values normalized for saturation induction B_max_. Red line—hysteresis measured along the ribbon (B_X_), blue line—hysteresis measured orthogonally, along the ribbon’s width (B_Y_). Slight differences in characteristics are evident, especially in the medium magnetizing field region (inset).

**Figure 5:**
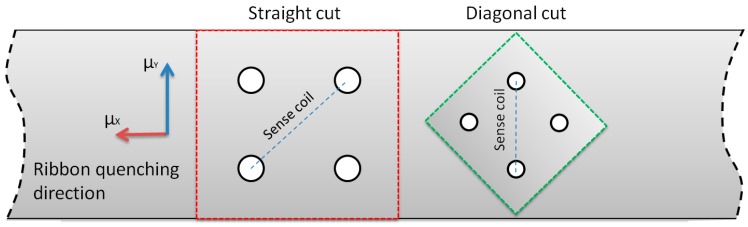
Schematic of ‘straight’ and ‘diagonal’ cut tensductor sensors.

The magnetic B(H) hysteresis curve of the ribbon was measured with Blacktower Ferrograph System (ESP, Warsaw, Poland). Single strips cut along and across the ribbon were used. Due to the slight magnetic anisotropy in the amorphous material (Figure 4), ‘straight’ cut sensors (Figure 5) proved to have non-monotonous characteristics, with minimal induced voltage for some significant loading force, and high zero-load output signal. It is possibly because the magnetizing and sensing coils are at 45° angle to the Y ribbon direction, and thus to the easy magnetization axis. The μ_X_ = μ_Y_, under no load condition is therefore lost. Alternatively, additional picked-up signal can be due to non-zero magnetostriction of the material, or even non-orthogonality error of magnetizing and sensing windings construction.

In order to overcome this problem, after an initial test with phase shifting of the measurement signal, ‘diagonal’ cut sensors were developed (Figure 5). The magnetizing coil is parallel to the ribbon length, thus the **H** field follows easy magnetization axis under no load condition, thus μ_X_ = μ_Y_.

The only disadvantage of the ‘diagonal’ sensor are its 1/$\sqrt{2}$ lower maximal dimensions for the given tape width, and hence lower measurement range.

The measurement range is limited by the durability of the sensor, which in turn depends on maximum allowable stress. In order to check the measurement range, destructive tests were performed by increasing load. The averaged stress was calculated from:

(4) $$\sigma =\frac{F}{A}$$

where: F is the tensile force, and A is the core ribbon cross section.

**Figure 6:**
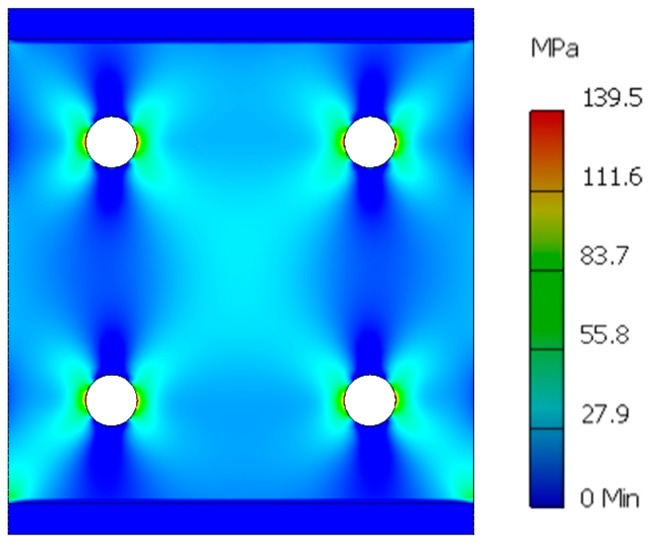
FEM calculation of force induced stresses in the tensed amorphous ribbon for 10 N load.

Nonmagnetic, rigid brass reinforcing bars were soldered to the two opposing ends of the core in order to evenly distribute the stresses in the core. FEM calculation of stress distribution for the core of utilized geometry and 10 N load is presented in Figure 6. As can be seen, there are stress concentrations near winding holes, and thus, these are the places where catastrophic failure can start during overload of the sensor.

The catastrophic failure occurs for distributed stresses between 40 MPa and 50 MPa, which is two-times assumed maximum measurement range. The range can be increased by increasing the core cross section, e.g., by using wider ribbon, or by stacking multiple ribbon layers together in standard transformer fashion. The maximum measurement range equation for the given geometry and material is therefore:

(5) $$F_{\mathrm{Max}}=\frac{\sigma_{\mathrm{Max}}}{2}A=\frac{\sigma_{\mathrm{Max}}}{2}n\;w\;t$$

where: F_Max_ is the maximum tensile force measurement range in N, A is the core ribbon cross section, σ_Max_ is the catastrophic failure limit stress in MPa, n is the number of core layers, w is the ribbon width in m, and t is the ribbon thickness in μm.

### 2.2. Measurement Test Stand

**Figure 7:**
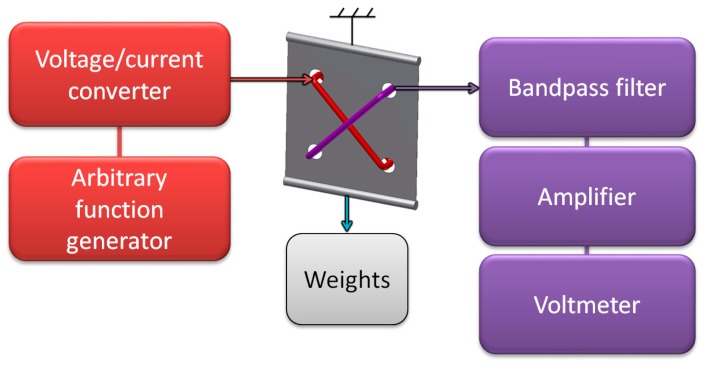
Schematic diagram of the measurement test stand.

The transducer operation was verified on specially constructed test stand, presented in Figure 7. Magnetizing winding was connected to the voltage/current converter RDM-2a (WUT, Warsaw, Poland), which was controlled by arbitrary function generator (SDG1025, Siglent, Helmond, The Netherlands). The measurement signal induced in sense coil is of the same frequency as in the magnetizing winding. Therefore, signal from sense winding was fed to the sharp band-pass filter and amplifier (Selective Nanovoltmeter 233, Unipan, Warsaw, Poland), the output of which was measured by digital voltmeter (TH1961, Tonghui, Changzhou, China). Force was set with the help of calibrated laboratory weights. First measurement stand was used with ‘straight’ sensor to investigate voltage vs. force characteristics, as well as second and third signal harmonic. 

**Figure 8:**
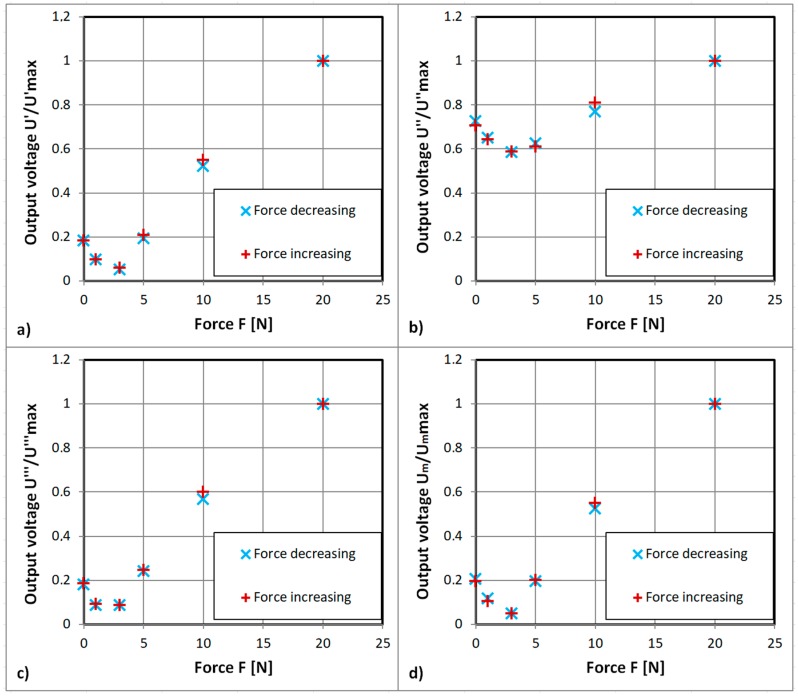
Obtained experimental characteristic of developed ‘straight’ sensor: (**a**) Normalized first harmonic of the sensor output voltage, for 1 kHz sinusoidal driving current of 500 mA, U′_max_ = 6.7 mV, (**b**) normalized second harmonic of the sensor output voltage, for 1 kHz sinusoidal driving current of 500 mA, U″_max_ = 0.96 mV, (**c**) normalized third harmonic of the sensor output voltage, for 1 kHz sinusoidal driving current of 500 mA, U‴_max_ = 3.9 mV, and (**d**) normalized first harmonic of the sensor output voltage, for 1 kHz sinusoidal driving current of 1000 mA with 5 Hz ramp down amplitude modulation, Um _max_ = 6.5 mV.

The initial experimental results for ‘straight’ cut sensor are presented in Figure 8. Results indicate that the ‘straight’ cut sensor has a null position at about 4 N of tensile force. It is probably due to the magnetic anisotropy of the amorphous tape being at 45° to the sensing winding.

Moreover, the first and third harmonic of the measurement signal (Figure 8a,c) have almost the same characteristics, but the amplitude of third harmonic is lower. The second harmonic (Figure 8b) is similar, with local minima for the same force, but the sensitivity is significantly lower. Therefore, all of the following characteristics were measured for the first harmonic only.

All of the presented characteristics exhibit hysteresis, which is a common problem for magnetoelastic technology [1,15]. Unfortunately, the characteristic for the ‘demagnetization’ modulated high driving current presented in Figure 8d is the same as for Figure 8a.

**Figure 9:**
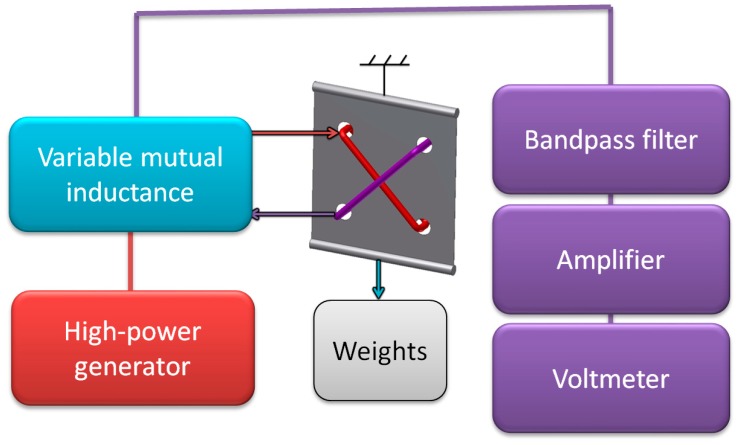
Schematic diagram of the improved measurement test stand.

Results from first test stand (presented in Figure 8) have caused modifications presented in Figure 9. Arbitrary function generator with U/I converter was substituted with high power audio frequency generator (Г3-32, Moscow, Russia). To achieve better force setting resolution with constant increment, steel bearing balls of 25.000 g were used as weights. The magnetizing and measurement signals were fed through variable mutual inductance, in order to phase shift the sensor characteristic, and achieve monotonous results for ‘straight’ sensor. 

**Figure 10:**
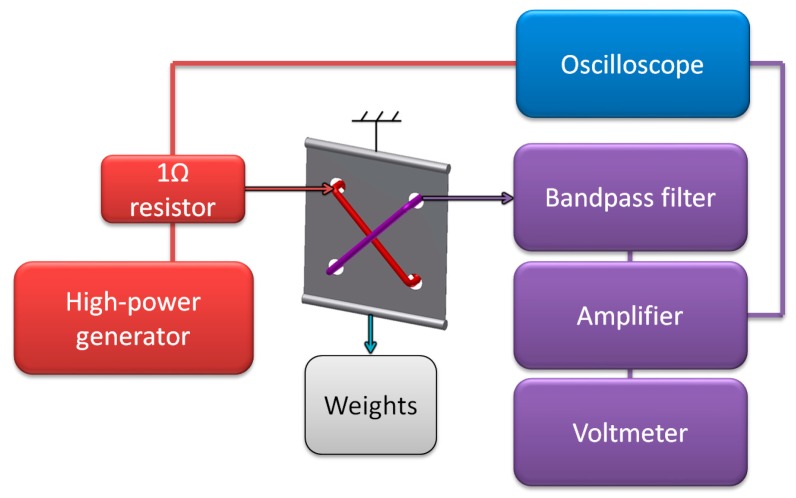
Schematic diagram of the final measurement test stand.

After careful consideration of sensor construction and obtained results, ‘diagonal’ sensor was developed, and the phase shifting mutual inductance was no longer needed. It was instead substituted for 1 Ω standard resistor, which allowed for magnetizing current waveform monitoring on oscilloscope (model 5228, Schlumberger/Sefram, St Etienne, France), together with filtered and amplified measurement signal (Figure 10). 

## 3. Results

**Figure 11:**
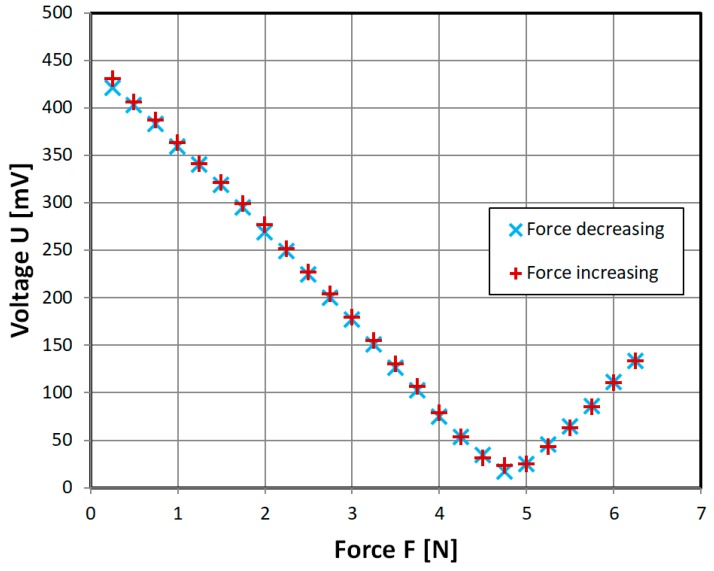
Obtained experimental characteristic of developed ‘straight’ sensor, with variable mutual inductance omitted. Sinusoidal driving current of 700 mA, 432 Hz.

The more detailed results for the ‘straight’ sensor obtained on the second measurement stand are presented in Figure 11 to investigate the local minimum. The unamplified signal was in the 1 mV amplitude region. Therefore, magnetizing current was set to a frequency of 432 Hz in order to decrease possible 50 Hz power line interference.

**Figure 12:**
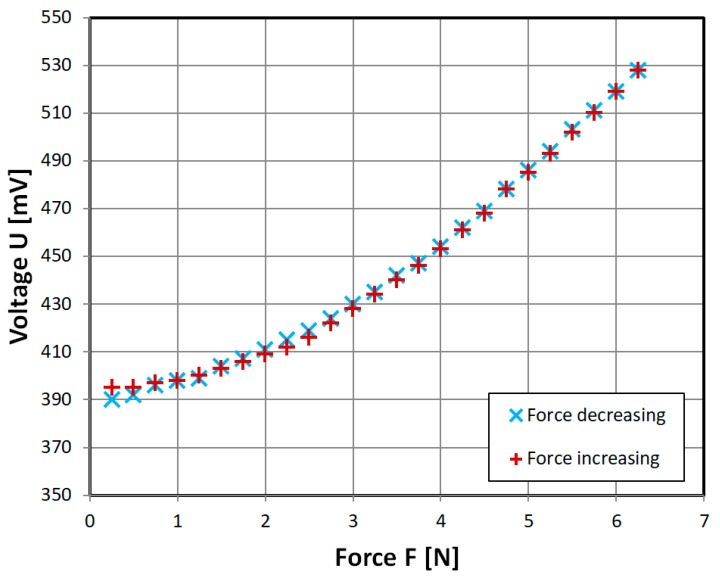
Obtained experimental characteristic of developed ‘straight’ sensor, with variable mutual inductance tuned for minimum at 0 N. Sinusoidal driving current of 700 mA, 432 Hz.

In Figure 12 experimental characteristic of ‘straight’ sensor with variable mutual inductance connected by to the magnetizing and sensing coils is shown. The variable inductance, of variometer type, was specially tuned while observing the measured signal to obtain minimal sensed voltage for 0 applied force, through interaction of magnetizing and phase shifted induced voltages. The obtained characteristic is indeed monotonous, but there is significant nonlinearity, and significant hysteresis in the low force region.

**Figure 13:**
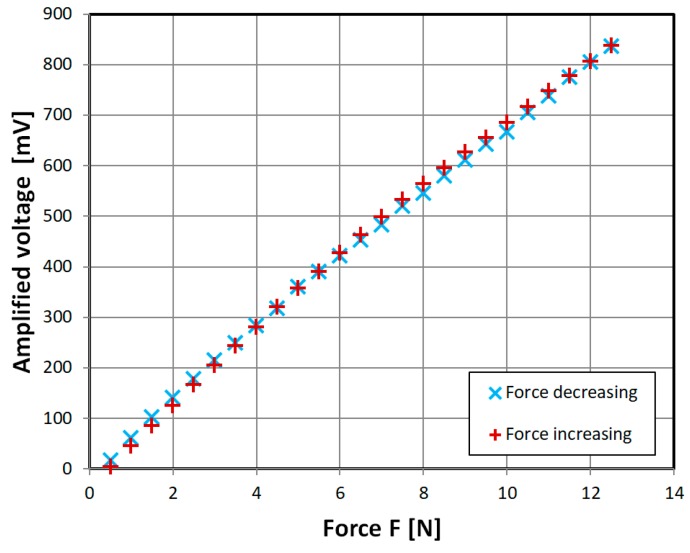
Obtained experimental characteristic of developed ‘diagonal’ sensor. Sinusoidal driving current of 1000 mA, 432 Hz.

In order to overcome the non-monotonous characteristic problem, and omit additional complication of mutual inductance, ‘diagonal’ cut sensor was developed. Its characteristic is almost linear (Figure 13), which allows for easy sensor implementation. There is also little hysteresis visible.

**Figure 14:**
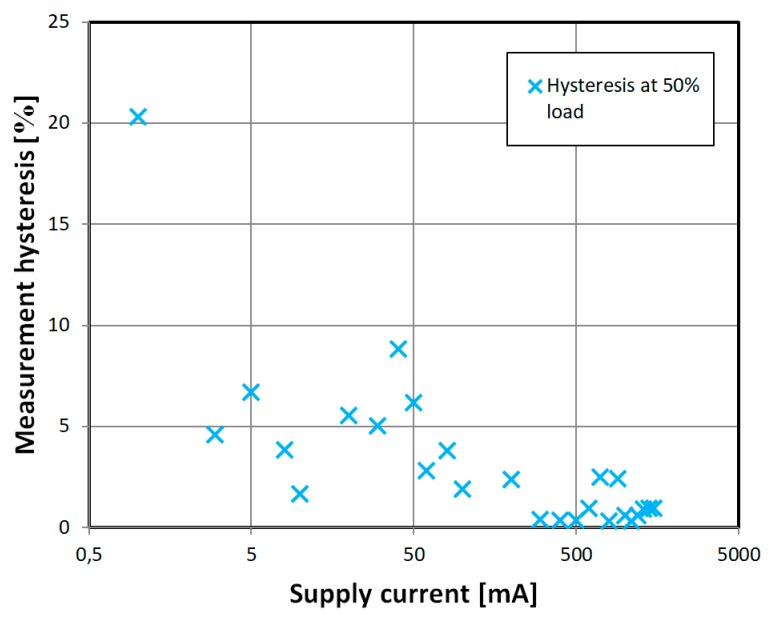
Obtained experimental characteristic of measurement hysteresis vs magnetizing current for the developed ‘diagonal’ sensor. Sinusoidal current of 432 Hz frequency.

The measurement hysteresis was investigated further for various amplitudes of magnetizing current (Figure 14). It was calculated as:

(6) $$e_{h}=\frac{{\left(x_{m}-x_{r}\right)}_{\max}}{x_{\max}-x_{\min}}$$

where: e_h_ is the measurement hysteresis, (x_m_ − x_r_)_max_—maximal width of measurement hysteresis loop (Figure 12), x_max_ is the maximal value reading, and x_min_ is the minimal value reading. It was found that the lowest hysteresis was in the 100 mA–1000 mA region, with some offshoots.

**Figure 15:**
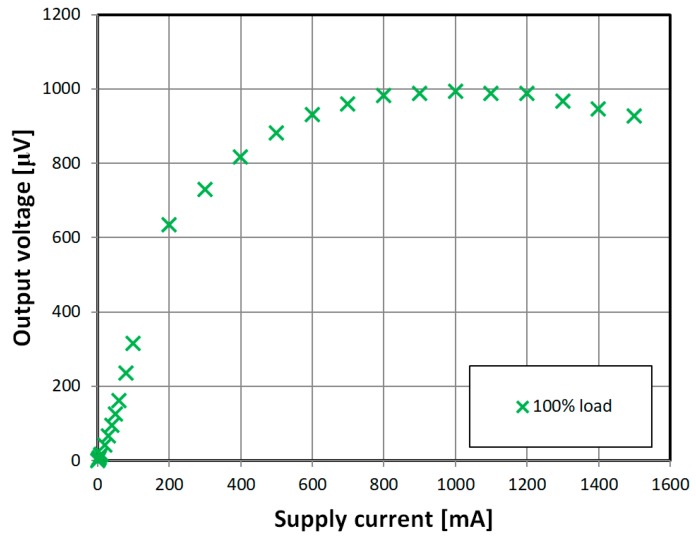
Obtained experimental characteristic of unamplified output voltage at 100% load (12 N) for ‘diagonal’ sensor for different magnetizing coil currents.

In the Figure 15 output voltage versus the supply magnetizing current characteristic of the ‘diagonal’ sensor is shown. Maximum in the characteristic shows beginning of magnetic saturation of the sample. Further decrease of the output is due to lengthening the time ratio of magnetic circuit working in saturated, low permeability region. Output voltage level could be raised with increase of sensing windings count or raising the operating frequency. For such low-level signals, proper band-pass filtering and amplification is essential.

## 4. Conclusions

The presented characteristic (Figure 13) is near-linear and monotonous, as opposed to recently published magnetoelastic sensors [16,17], which have nonlinear response, mostly due to Villari reversal point [9]. Measurement range is scalable with sensor geometry according to Equation (5), and allows for low-level force measurements. Its construction is relatively simple, which may compensate for average accuracy and measurement hysteresis, which is a common drawback of magnetoelastic sensors [1]. It also has greater stress-sensitivity and greater relative change of measurement signal than recent magnetoelastic sensors [17,18], but much lower maximum mechanical stress resistance of 50 MPa, as opposed to 200–3000 MPa for bulk metallic glasses [17]. The measurement hysteresis values are within typical range of magnetoelastic force sensors (0.5–3%) [1]. However, there are many methods of hysteresis compensation described in the literature [19,20,21], which could be employed in future works.

Presented work hints also on possibility of assessment of magnetic anisotropy in the utilized ribbons, e.g., with cores cut under different angles from the investigated tape. However, it would be rather laborious process. Therefore, future works will be focused on raising the accuracy of the presented sensor, as well as miniaturization and experiments with other highly magnetoelastic ferromagnetic ribbons.
